# A Patient with Systemic Lupus Erythematosus Complicated by Neurological Symptoms of Toluene Poisoning

**DOI:** 10.1155/2013/390960

**Published:** 2013-07-15

**Authors:** Kentaro Isoda, Tohru Takeuchi, Shigeki Makino, Toshiaki Hanafusa

**Affiliations:** Department of Internal Medicine (I), Osaka Medical College, 2-7 Daigaku-machi, Takatsuki, Osaka 569-8686, Japan

## Abstract

We report a patient with systemic lupus erythematosus complicated by toluene poisoning. She had erythema, alopecia, arthralgia, and various neurological symptoms. Laboratory findings showed leukocytopenia, low levels of complements, and anti-dsDNA antibody. However, normal interleukin-6 level and IgG index of cerebrospinal fluid and brain magnetic resonance imaging and single photon emission computed tomography findings suggested that her neurological symptoms were caused by metabolic disorder but not neuropsychiatric systemic lupus erythematosus. Erythema, alopecia, and arthralgia improved rapidly after administration of prednisolone and tacrolimus, whereas neurological symptoms improved only gradually. Because of a history of exposure to toluene, her neurological symptoms were considered to be due to toluene poisoning. The differentiation of toluene poisoning from neuropsychiatric systemic lupus erythematosus based on symptoms is difficult because both induce various neuropsychiatric disorders. Laboratory findings of cerebrospinal fluid, radiological findings, and medical interview were useful for differentiation of toluene poisoning from neuropsychiatric systemic lupus erythematosus.

## 1. Introduction

Systemic lupus erythematosus (SLE), a systemic inflammatory disease characterized by the presence of various autoantibodies, affects organs such as the skin, kidneys, and central nervous system. NPSLE includes diverse pathological conditions such as lesions in the central nervous system, spinal cord, and peripheral nerves due to angiitis, antiphospholipid syndrome, and hypercytokinemia and presents diverse neurological symptoms. Toluene is an organic solvent used to dilute paints and adhesives. It is fat soluble, volatile, and readily absorbed through the skin. Toluene inhibits synaptic transmission in the central nervous system and induces irreversible damage such as axonopathy and gliosis, resulting in the presentation of various neurological symptoms. Here, we report on a patient with active SLE complicated by toluene poisoning who presented with neuropsychiatric symptoms. Differentiation of neuropsychiatric symptoms between toluene poisoning and NPSLE was difficult in this case.

## 2. Case Report

**Figure 1:**
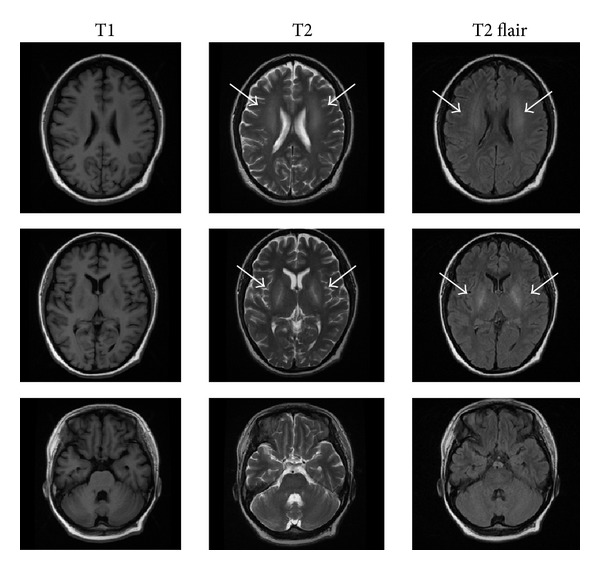
Brain MR imaging (T2WI·FLAIR) revealed bilateral symmetrical high signal intensities in the globus pallidi (arrows), cerebral hemispheres (triangles), and cerebral peduncles.

**Figure 2:**
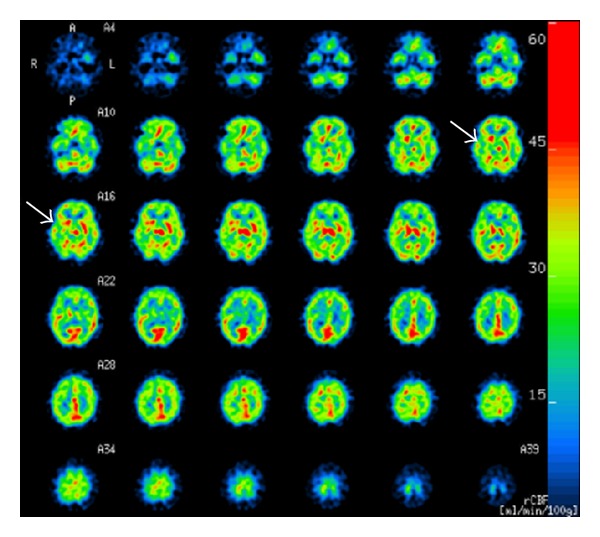
Brain perfusion scintigraphy (^123^I-IMP) showed a decrease in blood flow in the right lentiform nucleus (arrows) and bilateral symmetrical decreases in blood flow in the frontal, parietal, and temporal lobes.

**Figure 3:**
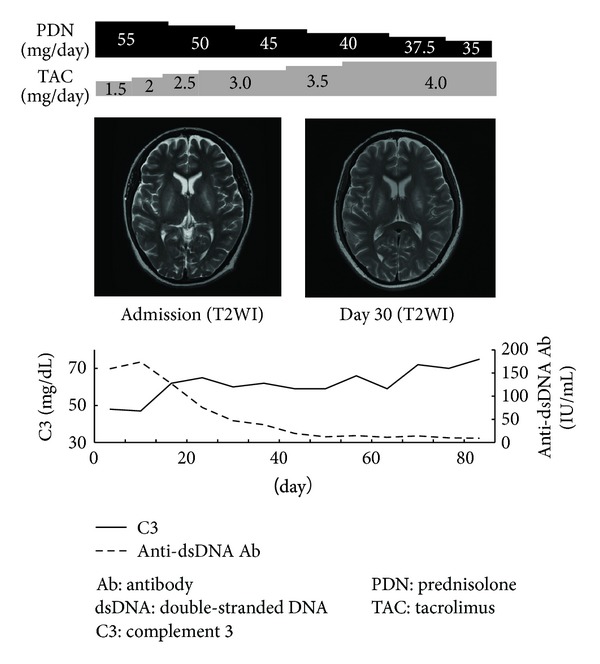
Clinical course: her symptoms such as erythema, alopecia, and arthralgia rapidly improved. Laboratory data showed increasing level of C3 and decreasing level of anti-double-stranded DNA antibody. Neuropsychiatric symptoms such as obnubilation, decreased muscle strength, and cerebellar ataxia generally improved. But her head MRI findings did not change anymore.

A 29-year-old woman noticed general malaise, abnormal gait, and epilation in January 2010 and, subsequently, decreased grip strength, livedo reticularis at the ends of her fingers, and stiffness of the bilateral knee joints. She had transient loss of consciousness with salivation, fatigability, and muscle pain around the shoulders in both May and June 2010 and visited and was admitted to our hospital. Photosensitivity, erythema on the bilateral forearms, alopecia, and arthralgia were observed. Laboratory findings showed leukocytopenia (2600/*μ*L), positive levels of antinuclear antibody (1 : 10240, shaggy pattern) and anti-double-stranded DNA antibody (190 IU/mL), and low level of complement-3 (48 mg/dL). The other disease-specific autoantibodies including antiphospholipid antibody were negative. A diagnosis of SLE was made on the basis of these findings [1]. Neurological examination showed obnubilation (Glasgow coma scale 14: E4, V4, A6), decreased muscle strength of the left upper limb (Manual Muscle Testing 3), and cerebellar ataxia such as wide based gait and dysdiadochokinesis. The interleukin-6 level and IgG index in cerebrospinal fluid (CSF) were 3.0 pg/mL (reference value <4.3 pg/mL) and 0.43 (reference value <0.6), respectively [1]. Head MRI (T2WI·FLAIR) revealed bilateral symmetrical high signal intensities in the globus pallidi, cerebral hemispheres, and cerebral peduncles (Figure 1). Brain perfusion scintigraphy (^123^I-IMP) showed a decrease in blood flow in the right lentiform nucleus and bilateral symmetrical decreases in blood flow in the frontal, parietal, and temporal lobes (Figure 2). These findings suggested that the neurological symptoms were due to other metabolic disorders but not to SLE. She made bags using an adhesive containing toluene in a small unventilated room alone (approximately 16 m^2^) and had worked about 60 hours a week over 21 months. On the basis of laboratory and radiological findings and clinical history, her neuropsychiatric symptoms were considered to be due to toluene poisoning. She had discontinued bag making one month before the admission. Her urinary hippuric acid level as an indicator of biological exposure to toluene was 0.235 g/g·Cr (reference value <1.6 g/g·Cr), within the normal range. Her SLE was treated with prednisolone 55 mg/day and tacrolimus 1.5 mg/day. The erythema, alopecia, and arthralgia rapidly improved with concomitant improvement of laboratory findings after immunosuppressive therapy. However, her neuropsychiatric symptoms such as disturbed consciousness, gait abnormality, cerebellar ataxia, and muscle weakness improved only gradually despite no changes in her head MRI findings (see Figure 3).

## 3. Discussion

We presented a case of SLE complicated by neurological symptoms of toluene poisoning. Both toluene poisoning and NPSLE induce various neuropsychiatric symptoms. Toluene poisoning causes higher brain dysfunction, cerebellar ataxia, pyramidal sign, involuntary movement, and peripheral neuropathy. NPSLE develops at the early stage of SLE, and headache, mood disorder, anxiety, and mild cognitive dysfunction are frequently observed [2]. Thus, the differentiation of these diseases based on symptoms is difficult. In this patient, interleukin-6 level and IgG index in CSF and the findings of head MRI and brain perfusion scintigraphy were not characteristic of NPSLE. Because there was a history of exposure to toluene, the cause of the neuropsychiatric symptoms was considered to be toluene poisoning.

Head MRI and brain perfusion scintigraphy are useful for differential diagnosis of NPSLE and other neurological disorders. In toluene poisoning, the characteristic findings of MRI are bilateral symmetrical high-intensity areas in the posterior limbs of the internal capsules and bilateral symmetrical low-intensity areas in the thalami on T2-weighted images [3]. Brain perfusion scintigraphy often reveals decreases in blood flow in the bilateral parietal, bilateral temporal, and left frontal lobes [4]. In NPSLE, T2-weighted MRI images of the head frequently show small patchy areas of high signal intensity scattering below the periventricular white matter [2]. Brain perfusion scintigraphy is more sensitive than MRI in NPSLE and shows multiple areas with decreased blood flow in the parietal and frontal lobes [5]. The findings of MRI and brain perfusion scintigraphy are reversible in NPSLE but not in toluene poisoning. Irreversible bilateral symmetrical lesions on head MRI T2WI·FLAIR and brain perfusion scintigraphy in this patient were consistent with those of toluene poisoning but not of NSLE.

Examination of CSF reveals increases in the cell count and protein in 40–50% of patients with NPSLE [2], and an increase in the IgG index reflecting immunoglobulin production has also been reported [6]. An increase (≥4.3 pg/mg) in interleukin-6 in CSF is useful for the diagnosis of lupus psychosis with a sensitivity of 87.5% and specificity of 92.3% [1]. Although the findings of CSF in toluene poisoning have not been reported, an elevation in the interleukin-6 level and IgG index in CSF was not observed in this patient.

There have been some reports on the association between SLE and chemical substances such as procainamide, isoniazid, and silica. In this context, there is a possibility that toluene poisoning induced the activity of SLE. However, there have been no reports on organic solvents including toluene, and the causal relation between toluene poisoning and SLE is unclear. 

We encountered a patient with SLE complicated by toluene poisoning who presented various neuropsychiatric symptoms. Neuropsychiatric symptoms of toluene poisoning resembled those of NPSLE. The findings of head MRI, brain perfusion scintigraphy, IL-6 and IgG index levels in the CSF, and medical interview are useful for the differentiation of toluene poisoning from NPSLE.
